# A comparison of pectoralis versus lumbar skeletal muscle indices for defining sarcopenia in diffuse large B-cell lymphoma - two are better than one

**DOI:** 10.18632/oncotarget.16552

**Published:** 2017-03-24

**Authors:** Se-Il Go, Mi Jung Park, Haa-Na Song, Hoon-Gu Kim, Myoung Hee Kang, Jung Hun Kang, Hye Ree Kim, Gyeong-Won Lee

**Affiliations:** ^1^ Division of Hematology-Oncology, Department of Internal Medicine, Gyeongsang National University Changwon Hospital, Gyeongsang National University College of Medicine, Changwon, Republic of Korea; ^2^ Department of Radiology, Gyeongsang National University Hospital, Gyeongsang National University College of Medicine, Jinju, Republic of Korea; ^3^ Division of Hematology-Oncology, Department of Internal Medicine, Gyeongsang National University Hospital, Gyeongsang National University College of Medicine, Jinju, Republic of Korea; ^4^ Institute of Health Science, Gyeongsang National University College of Medicine, Jinju, Republic of Korea

**Keywords:** sarcopenia, diffuse large B-cell lymphoma, muscle, drug toxicity, prognosis

## Abstract

**Backgrounds:**

Sarcopenia is known to be associated with poor clinical outcome in patients with diffuse large B-cell lymphoma (DLBCL). There is no consensus concerning the optimal method to define sarcopenia in DLBCL.

**Methods:**

We retrospectively reviewed 193 DLBCL patients treated with rituximab plus cyclophosphamide, doxorubicin, vincristine, and prednisone (R-CHOP) therapy. Sarcopenia was classified by the region where the pretreatment skeletal muscle index (SMI) was measured.

**Results:**

Both the sarcopenia-L3 and sarcopenia-pectoralis muscle (PM) groups had increased incidences of severe treatment-related toxicities and treatment discontinuation compared with the non-sarcopenia-L3 and non-sarcopenia-PM groups, respectively. The sarcopenia-L3 and non-sarcopenia-L3 groups had 5-year overall survival (OS) rates of 40.5% and 67.8% (*p* < 0.001), respectively. The sarcopenia-PM and non-sarcopenia-PM groups had 5-year OS rates of 35.9% and 69.0% (*p* < 0.001), respectively. When the sarcopenia-L3 alone and sarcopenia-PM alone groups were compared, there were no differences in baseline characteristics, treatment toxicity, or survival. In multivariate analysis, when compared with the non-sarcopenia-both group, OS was significantly worse in the sarcopenia-both group (HR, 2.480; 95% CI, 1.284 – 4.792; *p* = 0.007), but not in patients with either sarcopenia-L3 alone or sarcopenia-PM alone (*p* = 0.151).

**Conclusions:**

L3- and PM-SMIs are equally useful to define sarcopenia, which is related to intolerance to R-CHOP therapy and to worse survival in patients with DLBCL. More prognostic information can be obtained when these two SMIs are combined to define sarcopenia.

## INTRODUCTION

The introduction of rituximab into front-line therapy has dramatically improved the clinical outcome of patients with diffuse large B-cell lymphoma (DLBCL). Compared with conventional chemotherapy, rituximab-based regimens such as R-CHOP (rituximab plus cyclophosphamide, doxorubicin, vincristine, and prednisone) resulted in an increased complete response (CR) rate and prolonged survival, without a significant increase in toxicity, in patients with DLBCL [1–4]. However, it is debatable whether frail patients, who have an increased risk of treatment complications, benefit from R-CHOP therapy because pivotal randomized trials excluded these populations from the analysis [1, 2, 4]. Furthermore, DLBCL patients with significant comorbidities or poor performance status (PS) are intolerant to R-CHOP therapy and have poor prognosis, mainly due to treatment-related toxicities and frequent cessation of treatment [5–7]. Recent studies have suggested that alternative therapeutic strategies to the standard R-CHOP regimen, such as dose modification, reduction of chemotherapy cycles, and replacement of anthracycline with other agents, showed a favorable toxicity profile with acceptable survival rates in frail patients with DLBCL [8–12]. Therefore, clinical markers to identify patients who are intolerant to standard R-CHOP therapy need to be developed for individualized therapy in DLBCL patients.

Cancer cachexia is a multifactorial syndrome related to systemic inflammation and adverse outcomes in patients with cancer [13, 14]. Many studies have reported that inflammatory markers and other biomarkers for cancer cachexia can help to predict the prognosis and to improve the performance of prognostic indices in DLBCL [15–21]. Additionally, sarcopenia is known to be associated with an increased risk of treatment-related toxicity and a worse survival outcome in various solid tumors [22–26]. Unfavorable aspects of sarcopenia have also been evaluated in patients with DLBCL. In a French study of 82 elderly DLBCL patients treated with an R-CHOP or R-miniCHOP regimen, sarcopenic patients had a higher revised International Prognostic Index (R-IPI), more frequent discontinuation of treatment, and a higher risk of death than did non-sarcopenic patients [27]. In a Japanese study of 207 DLBCL patients who received R-CHOP or its derivative regimen, male – but not female – sarcopenic patients had worse progression-free survival (PFS) compared with non-sarcopenic patients [28]. These two previous studies used the L3 skeletal muscle index (L3-SMI), measured by computed tomography (CT), to determine sarcopenia. Recently, we demonstrated that sarcopenia, determined by pectoralis muscle SMI (PM-SMI), was also strongly associated with intolerance to treatment and with poor prognosis in DLBCL patients who were treated with the standard R-CHOP regimen [29]. In addition, we suggested that the addition of sarcopenic status to IPI components can improve the predictive power of IPI [29].

In this study, given the uncertainty about the optimal SMI to define clinically meaningful sarcopenia in DLBCL, we compared the characteristics and clinical outcome between sarcopenic patients determined by L3-SMI and those determined by PM-SMI who were treated with standard front-line R-CHOP therapy. Furthermore, the synergistic role of L3- and PM-SMIs as prognostic markers was also investigated.

## RESULTS

### Patient population and characteristics

**Figure 1:**
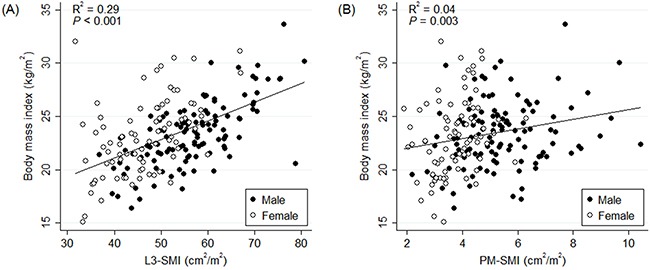
Correlation between body mass index and **(A)** L3-SMI and **(B)** PM-SMI. Abbreviations: L3-SMI = L3 skeletal muscle index, PM-SMI = pectoralis muscle skeletal muscle index.

In total, 193 patients were included in the final analysis. There were 116 patients in the non-sarcopenia-both group. Of the remaining 77 patients with some form of sarcopenia, 22, 30, and 25 patients were classified into the sarcopenia-both, sarcopenia-L3 alone, and sarcopenia-PM alone groups, respectively. When the sarcopenic status was dichotomized according to the type of SMI, 52 and 141 patients comprised the sarcopenia-L3 and non-sarcopenia-L3 groups, and 55 and 138 patients comprised the sarcopenia-PM and non-sarcopenia-PM groups, respectively. There were no differences in L3-SMI between non-sarcopenia-both and sarcopenia-PM alone groups (*p* = 0.132) and in PM-SMI between non-sarcopenia-both and sarcopenia-L3 alone groups (*p* = 0.293). Both L3- and PM-SMIs were significantly and weakly associated with BMI (R^2^ = 0.29, *p* < 0.001 and R^2^ = 0.04, *p* = 0.003, respectively; Figure 1).

Baseline characteristics of patients are presented in Table 1. Compared with the non-sarcopenia-L3 group, the sarcopenia-L3 group was associated with old age, poor PS, B-symptoms, advanced Ann Arbor stage, higher IPI, R-IPI, and National Comprehensive Cancer Network-IPI (NCCN-IPI), and hypoalbuminemia. The sarcopenia-PM group also had the adverse clinical characteristics observed in the sarcopenia-L3 group, except PS and Ann Arbor stage. When the sarcopenia-L3 alone and sarcopenia-PM alone groups were directly compared, there were no differences in any of the baseline characteristics.

**Table 1:** Patients’ characteristics and treatment response

	Sarcopenia-both(n = 22)	Sarcopenia-L3/PM alone (n = 55)		Non-sarcopenia-both(n = 116)	*P*^†^	*P*^‡^	*P*_trend_^§^
		L3 alone(n = 30)	PM alone(n = 25)				
Age, years					0.001	0.017	0.002
Median	68	66	65	58.5			
Range	47 – 81	27 – 86	24 – 76	21 – 82			
Sex					0.113	0.805	0.243
Male	16 (72.7)	19 (63.3)	12 (48.0)	65 (56.0)			
Female	6 (27.3)	11 (36.7)	13 (52.0)	51 (44.0)			
ECOG PS					< 0.001	0.056	0.001
0 – 1	11 (50.0)	17 (56.7)	18 (72.0)	94 (81.0)			
2 – 3	11 (50.0)	13 (43.3)	7 (28.0)	22 (19.0)			
B-symptoms					0.037	0.003	0.001
Absent	12 (54.5)	24 (80.0)	18 (72.0)	99 (85.3)			
Present	10 (45.5)	6 (20.0)	7 (28.0)	17 (14.7)			
Bulky disease					0.141	0.058	0.034
Non-bulky	15 (68.2)	25 (83.3)	20 (80.0)	101 (87.1)			
Bulky	7 (31.8)	5 (16.7)	5 (20.0)	15 (12.9)			
Ann Arbor stage					0.015	0.158	0.015
I – II	5 (22.7)	11 (36.7)	12 (48.0)	59 (50.9)			
III – IV	17 (77.3)	19 (63.3)	13 (52.0)	57 (49.1)			
Extranodal involvement					0.012	0.092	0.008
0 – 1 site	12 (54.5)	17 (56.7)	16 (64.0)	89 (76.7)			
> 1 site	10 (45.5)	13 (43.3)	9 (36.0)	27 (23.3)			
LDH					0.153	0.454	0.168
Normal	8 (36.4)	10 (33.3)	10 (40.0)	55 (47.4)			
Elevated	14 (63.6)	20 (66.7)	15 (60.0)	61 (52.6)			
IPI					0.003	0.006	< 0.001
Low to low-intermediate	6 (27.3)	15 (50.0)	13 (52.0)	77 (66.4)			
High-intermediate to high	16 (72.7)	15 (50.0)	12 (48.0)	39 (33.6)			
R-IPI					0.003	0.014	< 0.001
Very good	0 (0.0)	2 (6.7)	3 (12.0)	25 (21.6)			
Good	6 (27.3)	13 (43.3)	10 (40.0)	52 (44.8)			
Poor	16 (72.7)	15 (50.0)	12 (48.0)	39 (33.6)			
NCCN-IPI					0.011	0.013	0.002
Low to low-intermediate	5 (22.7)	13 (43.3)	11 (44.0)	67 (57.8)			
High-intermediate to high	17 (77.3)	17 (56.7)	14 (56.0)	49 (42.2)			
Albumin					< 0.001	< 0.001	< 0.001
Normal	6 (27.3)	18 (60.0)	12 (48.0)	94 (81.0)			
Hypoalbuminemia	16 (72.7)	12 (40.0)	13 (52.0)	22 (19.0)			
Treatment response					0.006	0.002	< 0.001
CR	11 (50.0)	20 (66.7)	16 (64.0)	99 (85.3)			
PR, NR/SD, or PD	7 (31.8)	9 (30.0)	6 (24.0)	12 (10.3)			
Not available*	4 (18.2)	1 (3.3)	3 (12.0)	5 (4.3)			

Data are presented as number of patients (%) except age.

There were no statistical differences between sarcopenia-L3 alone and -PM alone groups (data not shown).

Abbreviations: PM = pectoralis muscle, ECOG PS = Eastern Cooperative Oncology Group performance status, LDH = lactate dehydrogenase, IPI = International Prognostic Index, R-IPI = revised International Prognostic Index, NCCN-IPI = National Comprehensive Cancer Network International Prognostic Index, CR = complete response, PR = partial response, NR = no response, SD = stable disease, PD = progressive disease.

*Information for treatment response was not available in 13 patients due to the following reasons: early discontinuation of treatment due to treatment toxicity after the first cycle of chemotherapy (6 patients); patient's own will to withdraw the treatment (5 patients); and loss of follow-up (2 patients).

†Comparing sarcopenia-L3 (sarcopenia-both + sarcopenia-L3 alone) with non-sarcopenia-L3 (others).

‡Comparing sarcopenia-PM (sarcopenia-both + sarcopenia-PM alone) with non-sarcopenia-PM (others).

§Comparing sarcopenia-both with sarcopenia-L3/PM alone and non-sarcopenia-both.

It was found that the greater the sarcopenic status, the worse the clinical status of patients. Considering baseline characteristics such as age, PS, tumor stage, and prognostic indices, the sarcopenia-both group had the worst clinical features, while the sarcopenia-L3/PM alone group had intermediate clinical features and the non-sarcopenia-both group had the most favorable clinical features.

### Treatment-related toxicity and compliance

Comparisons of toxicity and compliance for R-CHOP therapy between groups are described in Table 2. Compared with the non-sarcopenia-L3 group, the sarcopenia-L3 group had more frequent grade 3 anemia (28.9% vs. 14.9%, *p* = 0.027), grade 3–4 (34.6% vs. 18.4%, *p* = 0.017) and grade 4 (23.1% vs. 10.6%, *p* = 0.027) thrombocytopenia, grade 4–5 non-hematologic toxicity (21.2% vs. 5.7%, *p* = 0.001), TRM (23.1% vs. 3.6%, *p* < 0.001), and treatment discontinuation (34.6% vs. 13.5%, *p* = 0.001). Similarly, the sarcopenia-PM group had more frequent grade 3 anemia (38.3% vs. 12.3%, *p* < 0.001), grade 3–4 (36.2% vs. 18.5%, *p* = 0.012) and grade 4 (23.4% vs. 11.0%, *p* = 0.032) thrombocytopenia, grade 4–5 non-hematologic toxicity (19.2% vs. 6.9%, *p* = 0.022), TRM (19.2% vs. 5.5%, *p* = 0.007), and treatment discontinuation (29.8% vs. 15.8%, *p* = 0.034), in addition to febrile neutropenia (grade 3–4, 48.9% vs. 28.1%, *p* = 0.008; grade 4–5, 23.4% vs. 9.6%, *p* = 0.014), compared to the non-sarcopenia-PM group. However, there were statistically no differences in treatment-related toxicity or treatment discontinuation between the sarcopenia-L3 alone and sarcopenia-PM alone groups.

**Table 2:** Comparison of toxicity and compliance for R-CHOP therapy according to sarcopenic status

	Sarcopenia-both(n = 22)	Sarcopenia-L3/PM alone (n = 55)		Non-sarcopenia-both(n = 116)	*P*^†^	*P*^‡^	*P*_trend_^§^
		L3 alone(n = 30)	PM alone(n = 25)				
Grade 3–5 hematologic toxicity							
Anemia (G3)	9 (40.9)	6 (20.0)	9 (36.0)	12 (10.3)	0.027	< 0.001	< 0.001
Neutropenia (G3–4)	19 (86.4)	25 (83.3)	19 (76.0)	99 (85.3)	0.876	0.508	0.756
Neutropenia (G4)	19 (86.4)	22 (73.3)	18 (72.0)	82 (70.7)	0.271	0.314	0.184
Thrombocytopenia (G3–4)	11 (50.0)	7 (23.3)	6 (24.0)	20 (17.2)	0.017	0.012	0.002
Thrombocytopenia (G4)	7 (31.8)	5 (16.7)	4 (16.0)	11 (9.5)	0.027	0.032	0.006
Febrile neutropenia (G3–5)	13 (59.1)	7 (23.3)	10 (40.0)	34 (29.3)	0.342	0.008	0.025
Febrile neutropenia (G4–5)	6 (27.3)	4 (13.3)	5 (20.0)	10 (8.6)	0.115	0.014	0.011
G3–5 non-hematologic toxicity	10 (45.5)	14 (46.7)	10 (40.0)	35 (30.2)	0.067	0.263	0.062
G4–5 non-hematologic toxicity	7 (31.8)	4 (13.3)	2 (8.0)	6 (5.2)	0.001	0.022	< 0.001
Treatment-related mortality	7 (31.8)	5 (16.7)	2 (8.0)	3 (2.6)	< 0.001	0.007	< 0.001
Treatment discontinuation*	9 (40.9)	9 (30.0)	5 (20.0)	14 (12.1)	0.001	0.034	0.001

Data are presented as number of patients having an event (%).

There were no statistical differences between sarcopenia-L3 alone and -PM alone groups (data not shown).

*Three patients discontinued the treatment after 3 cycles of R-CHOP due to early disease progression.

†Comparing sarcopenia-L3 (sarcopenia-both + sarcopenia-L3 alone) with non-sarcopenia-L3 (others).

‡Comparing sarcopenia-PM (sarcopenia-both + sarcopenia-PM alone) with non-sarcopenia-PM (others).

§Comparing sarcopenia-both with sarcopenia-L3/PM alone and non-sarcopenia-both.

The sarcopenia-both group was extremely intolerant to R-CHOP therapy. The rates of grade 3 anemia (40.9%), grade 3–4 (50.0%) and grade 4 (31.8%) thrombocytopenia, grade 3–5 (59.1%) and grade 4–5 (27.3%) febrile neutropenia, grade 4–5 non-hematologic toxicity (31.8%), TRM (31.8%), and treatment discontinuation (40.9%) were highest in the sarcopenia-both group, followed by the sarcopenia-L3/PM alone group and the non-sarcopenia-both group.

### Treatment response

Data for treatment response were available for 180 of 193 patients (Table 1). In the analysis of all 193 patients, the CR rate was much lower in the sarcopenia-both group than in the sarcopenia-L3/PM alone group and in the non-sarcopenia-both group (50.0% vs. 65.5% vs. 85.3%, *p* < 0.001). When treatment response was assessed in 159 patients, excluding 34 patients who discontinued treatment due to reasons other than disease progression, the difference in the CR rate was still significant between the sarcopenia-both, sarcopenia-L3/PM alone, and non-sarcopenic groups, but the significance was lower (71.4% vs. 83.3% vs. 92.2%, *p* = 0.043). There was no difference in the CR rate between the sarcopenia-L3 alone and sarcopenia-PM alone groups (*p* = 0.511).

### Survival

**Figure 2:**
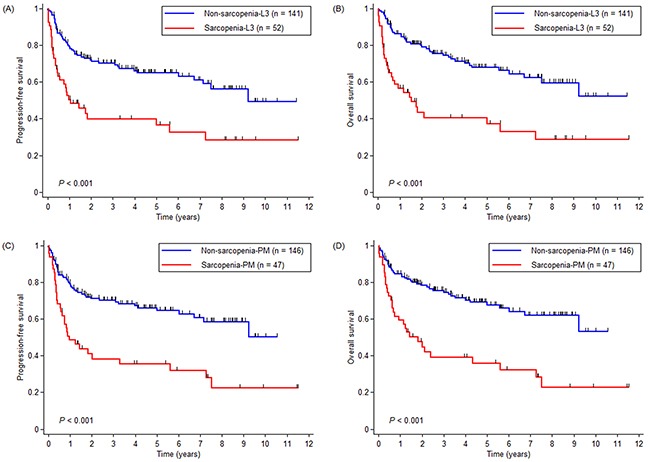
Kaplan-Meier curves for **(A and C)** progression-free survival and **(B and D)** overall survival (OS) according to sarcopenic status determined by L3-SMI and by PM-SMI, respectively. Abbreviations: L3-SMI = L3 skeletal muscle index, PM-SMI = pectoralis muscle skeletal muscle index.

The median follow-up durations were 58.4 and 52.4 months in all patients and in survivors, respectively. The sarcopenia-L3 group had worse PFS (5-year PFS, 39.8% vs. 64.9%, *p* < 0.001; Figure 2A) and OS (5-year OS, 40.5% vs. 67.8%, *p* < 0.001; Figure 2B) than did the non-sarcopenia-L3 group. The sarcopenia-PM group also had longer PFS (5-year PFS, 35.5% vs. 66.0%, *p* < 0.001; Figure 2C) and OS (5-year OS, 35.9% vs. 69.0%, *p* < 0.001; Figure 2D), compared with the non-sarcopenia-PM group.

**Figure 3:**
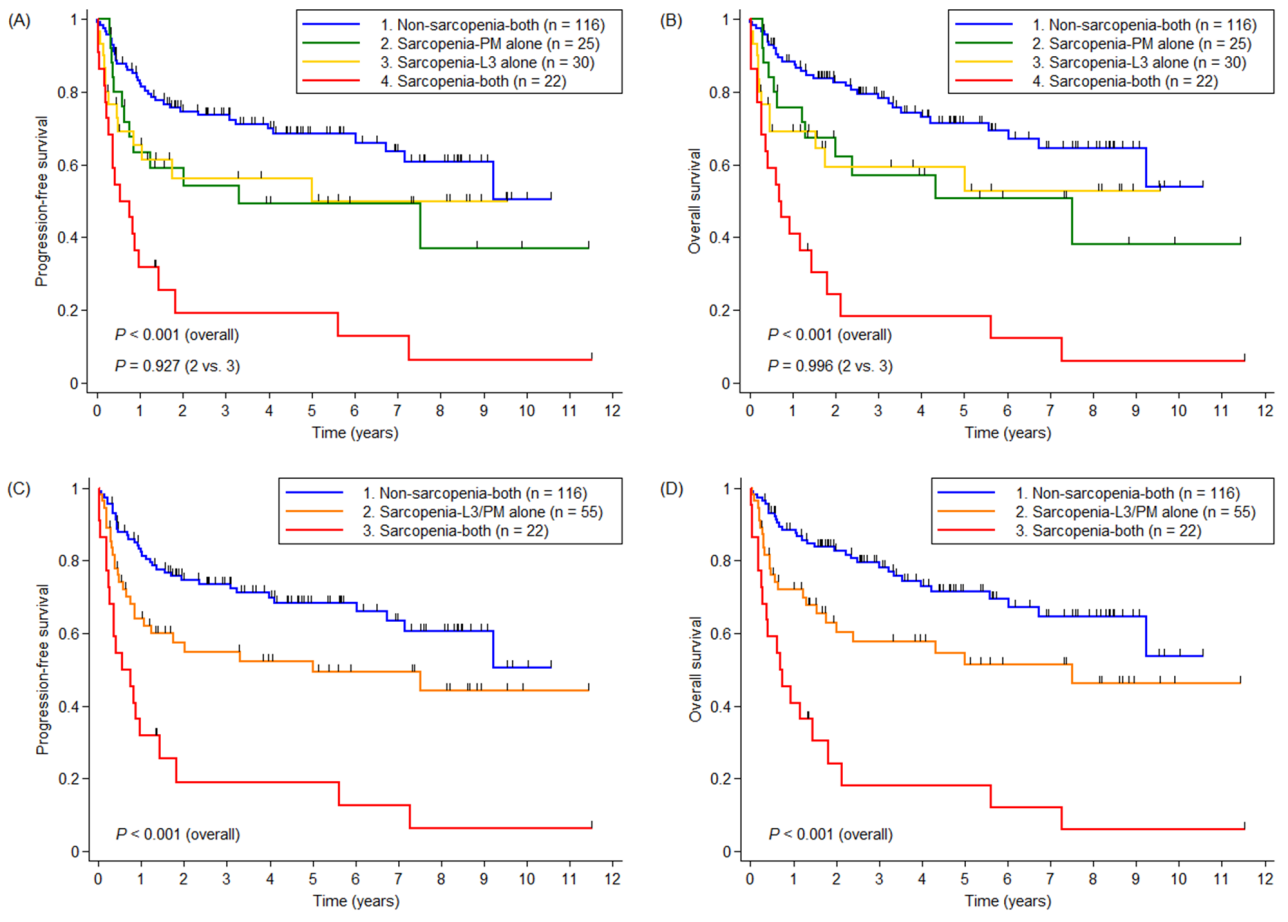
Kaplan-Meier curves for **(A and C)** progression-free survival and **(B and D)** OS in three and four groups divided according to sarcopenic status, respectively. Abbreviations: PM = pectoralis muscle.

When the sarcopenia-L3 alone and sarcopenia-PM alone groups were compared, there were no differences in PFS (*p* = 0.927; Figure 3A) or OS (*p* = 0.996; Figure 3B). Given the very similar survival curves of the sarcopenia-L3 alone and sarcopenia-PM alone groups, the sarcopenia-L3/PM alone group, which comprised these two groups, was used in further survival analyses. The sarcopenia-both group had the worst PFS (5-year PFS, 19.1% vs. 52.4% vs. 68.5%, *p* < 0.001; Figure 3C) and OS (5-year OS, 18.2% vs. 54.5% vs. 71.5%, *p* < 0.001; Figure 3D), followed by the sarcopenia L3/PM alone group and the non-sarcopenia-both group.

In multivariate analysis adjusted for B-symptoms, albumin status, and five factors of the IPI, having both types of sarcopenia (i.e., the sarcopenia-both group but not the sarcopenia-L3/PM alone group), was one of the independent factors for worse PFS (HR = 2.166; 95% CI: 1.146 – 4.095; *p* = 0.017) and OS (HR = 2.480; 95% CI: 1.284 – 4.792; *p* = 0.007), compared with the non-sarcopenia-both group (Table 3).

**Table 3:** Univariate and multivariate analyses for PFS and OS

Factor	PFS						OS					
	Univariate			Multivariate			Univariate			Multivariate		
	HR	95% CI	*p*	HR	95% CI	*p*	HR	95% CI	*p*	HR	95% CI	*p*
Age, years												
≤ 60	Ref.			Ref.			Ref.			Ref.		
> 60	3.092	1.878 – 5.089	< 0.001	1.826	1.056 – 3.159	0.031	3.246	1.925 – 5.475	< 0.001	1.962	1.108 – 3.476	0.021
Sex												
Male	Ref.						Ref.					
Female	0.803	0.514 – 1.256	0.337				0.802	0.502 – 1.281	0.355			
ECOG PS												
0 – 1	Ref.			Ref.			Ref.			Ref.		
2 – 3	3.525	2.255 – 5.511	< 0.001	2.023	1.205 – 3.396	0.008	3.883	2.434 – 6.196	< 0.001	2.145	1.248 – 3.687	0.006
B-symptoms												
Absent	Ref.			Ref.			Ref.			Ref.		
Present	2.607	1.639 – 4.149	< 0.001	1.241	0.724 – 2.125	0.432	2.589	1.596 – 4.199	< 0.001	1.175	0.679 – 2.032	0.565
Bulky disease												
Non-bulky	Ref.						Ref.					
Bulky	0.744	0.393 – 1.410	0.365				0.827	0.435 – 1.574	0.563			
Ann Arbor stage												
I – II	Ref.			Ref.			Ref.			Ref.		
III – IV	3.905	2.342 – 6.510	< 0.001	2.192	1.125 – 4.272	0.021	3.665	2.162 – 6.213	< 0.001	2.094	1.041 – 4.212	0.038
Extranodal involvement												
0 – 1 site	Ref.			Ref.			Ref.			Ref.		
> 1 site	4.213	2.690 – 6.599	< 0.001	1.816	1.018 – 3.239	0.043	3.939	2.465 – 6.295	< 0.001	1.715	0.929 – 3.166	0.084
LDH												
Normal	Ref.			Ref.			Ref.			Ref.		
Elevated	2.596	1.596 – 4.221	< 0.001	1.454	0.845 – 2.501	0.176	2.882	1.720 – 4.829	< 0.001	1.702	0.955 – 3.032	0.071
Albumin												
Normal	Ref.			Ref.			Ref.			Ref.		
Hypoalbuminemia	3.281	2.119 – 5.082	< 0.001	1.345	0.810 – 2.234	0.252	3.873	2.445 – 6.134	< 0.001	1.526	0.896 – 2.600	0.120
BMI												
Normal to obese	Ref.						Ref.					
Underweight	1.553	0.676 – 3.569	0.300				1.749	0.759 – 4.033	0.190			
Sarcopenia status												
Non-sarcopenia-both	Ref.			Ref.			Ref.			Ref.		
Sarcopenia-L3/PM alone	1.785	1.080 – 2.951	0.024	1.401	0.820 – 2.393	0.218	1.927	1.134 – 3.275	0.015	1.512	0.860 – 2.658	0.151
Sarcopenia-both	4.836	2.764 – 8.462	< 0.001	2.166	1.146 – 4.095	0.017	5.808	3.265 – 10.332	< 0.001	2.480	1.284 – 4.792	0.007

Abbreviations: PFS = progression-free survival, OS = overall survival, HR = hazard ratio, CI = confidence interval, ECOG PS = Eastern Cooperative Oncology Group performance status, LDH = lactate dehydrogenase, BMI = body mass index, PM = pectoralis muscle.

## DISCUSSION

Cancer-associated cachexia consumes skeletal muscle in cancer patients through the complex mechanisms. An experimental study suggested that adipose triglyceride lipase leads to sarcopenia by causing complete loss of white adipose tissue [14]. Another study reported that IL-6 mediates sarcopenia in cancer-associated cachexia by activating FOXO3 and atrogin [30]. Previous clinical studies reported that sarcopenia leads to higher drug exposure, which results in an increased incidence of dose-limiting toxicities in patients with cancer [31–33]. Similarly, the present study showed that sarcopenia was related to severe treatment-related toxicities, frequent withdrawal from treatment with consequential poor response rate, and worse survival in patients with DLBCL. Sarcopenia was also associated with factors indicative of poor nutritional status such as hypoalbuminemia and low BMI. These findings were identical in the sarcopenia-L3 and sarcopenia-PM groups. Furthermore, there were no differences in any clinical outcome between the sarcopenia-L3 alone and sarcopenia-PM alone groups.

An international consensus for cancer cachexia proposes to use lumbar SMI determined by CT to define sarcopenia [13]. In fact, numerous studies have used L3-SMI to determine sarcopenia [24, 26–28, 33–36]. However, a recent study reported that the cross-sectional area of the pectoralis muscle determined by low-dose chest CT scan was significantly correlated with total body skeletal muscle mass, as measured by bioelectrical impedance analysis in healthy subjects [37]. The clinical importance of sarcopenia determined by the pectoralis muscle was assessed in respiratory tract disorders such as chronic obstructive pulmonary disease (COPD) [38] and small cell lung cancer (SCLC) [25]. COPD patients with lower pectoralis muscle area had more severe airflow obstruction, worse exercise capacity, and poorer quality of life than those with higher pectoralis muscle area [38]. Male SCLC patients with sarcopenia determined by PM-SMI were likely to have more TRM, frequent early discontinuation of treatment, and poor prognosis compared with those without sarcopenia [25]. Taken together with these previous reports, the present study more strongly supports our previous finding [29] that sarcopenia determined by PM-SMI has an adverse impact in patients with DLBCL. There is another advantage in using the pectoralis muscle to determine sarcopenia in that it can be easily measured due to its anatomical simplicity [37, 38].

In subsequent analyses, we found that the extent of sarcopenia could be classified more specifically when both L3- and PM-SMIs were used together to determine sarcopenia. Over 30% and 40% of the patients who met the criteria for sarcopenia determined by both L3-SMI and PM-SMI experienced TRM and had early treatment discontinuation, respectively, in the present study. These patients had a 2.5 times higher risk of death in multivariate analysis adjusted for well-known prognostic factors, compared to patients who met neither the criteria for sarcopenia determined by L3-SMI nor those determined by PM-SMI. In contrast, patients who met only one of the criteria showed features intermediate between the sarcopenia-both and non-sarcopenia-both groups in terms of treatment-related toxicity and compliance with treatment. Furthermore, their survival and level of SMIs which did not meet the criteria for sarcopenia (e.g. L3-SMI in sarcopenia-PM alone group) was statistically not different with those of non-sarcopenia-both group. These findings suggest that PM-SMI is not only an alternative to L3-SMI but also has a complementary role to L3-SMI in determining sarcopenia. Several previous studies also proposed prognostic models that combine sarcopenia and other clinical factors relevant to cachexia. When a score combining sarcopenia and hypoalbuminemia replaced sarcopenia alone as a prognostic factor in DLBCL, HR for OS increased from 2.07 (sarcopenia alone) to 3.53 (higher score) [27]. Another study performed in the same cohort showed that sarcopenia and adipopenia can be integrated to predict the prognosis more accurately, when compared to the use of sarcopenia alone [39]. In metastatic renal cell cancer, sarcopenic patients with lower BMI experienced more dose-limiting toxicities from sunitinib therapy [26]. All of these findings imply that a more comprehensive approach is needed to identify cachectic patients, instead of identifying them using only sarcopenia determined by L3-SMI alone. In addition, given that chest and abdominal CT are routinely used in the initial assessment of patients with DLBCL, our approach using both L3 -and PM-SMIs to determine sarcopenic status is easily applicable in clinical practice.

There is a debate regarding whether sarcopenia is directly associated with poor response to anticancer therapy. Sarcopenia results in a higher plasma concentration of anticancer drugs [31, 32], which may be related to better response to treatment [40]. A higher rate of pathologic CR was reported in sarcopenic patients compared with non-sarcopenic patients who were treated with neoadjuvant chemotherapy for breast cancer [41]. However, early discontinuation of treatment owing to severe toxicity may limit the efficacy of anticancer therapy in patients with sarcopenia [40]. In patients who received neoadjuvant chemotherapy for esophageal cancer, the presence of sarcopenia did not affect the rate of pathological chemotherapy response [42]. In SCLC, objective response rate was not different between sarcopenic and non-sarcopenic patients if patients who experienced early discontinuation of treatment before first response evaluation were excluded from the analysis [25]. In the present study, treatment response became worse according to the degree of sarcopenia. However, the difference in treatment response became less marked, with borderline significance after patients who discontinued treatment due to potential treatment toxicity or non-compliance were excluded from the analysis. Therefore, we suggest that intensive supportive care may be necessary to maximize and maintain the efficacy of anticancer therapy in sarcopenic patients.

The main limitation of the present study was its retrospective nature. This makes the results rather difficult to interpret because of potentially inaccurate data collection, selection bias between groups, and missing of clinically important information such as the cell-of-origin subtype. Although the relationship between the sarcopenic status and the cell-of-origin has still not been confirmed, a previous report showed that there was no association between these two factors in patients with DLBCL [27]. Another limitation is a small sample size, which may limit the generalizability of findings. Therefore, the results observed in the present study should be validated in prospective studies to overcome the problems described above.

In conclusion, we demonstrated that both L3- and PM-SMIs can be equally used to define sarcopenia, which is associated with intolerance to R-CHOP therapy and poor prognosis in DLBCL patients. The use of only one muscle index may not be enough to define sarcopenia. Also, we expect that, when these two muscle indices are considered together to assess sarcopenic status, treatment in DLBCL patients can be more individualized.

## MATERIALS AND METHODS

### Patients

We retrospectively reviewed all consecutive DLBCL patients who were treated with the standard front-line R-CHOP regimen between January 2004 and October 2015 at Gyeongsang National University Hospital (GNUH). Among them, patients whose baseline CT chest and abdomen scans were available were included in this study. Exclusion criteria were as follows: 1) younger than 18 years of age, 2) front-line therapy other than the R-CHOP regimen, and 3) transformation from another type of lymphoma. This study was approved by the Institutional Review Board of GNUH.

### Muscle mass measurement

CT scans were performed using a 64-detector CT (Brilliance-64; Philips Medical Systems, Best, The Netherlands) with a detector configuration of 64 × 0.625 mm, a tube voltage of 120 kVp, a fixed tube current of 200 mAs, a pitch of 0.923, a gantry rotation time of 0.5 s, and a smooth reconstruction (Philips “B”) filter. In the chest CT, the whole lung was scanned, from the lung apex to the diaphragm. In the abdominal CT, the whole abdomen was scanned, from the diaphragmatic dome to the pubic symphysis. The mass of the pectoralis muscle, including the pectoralis major and minor, and the muscle mass of the L3 region, including the abdominal wall, psoas, and paraspinal muscles, were measured by one radiologist with 8 years of experience.

The measurement method was as follows. First, reconstructed axial images with a 3-mm slice thickness and 3-mm interval were analyzed at the levels of the fourth thoracic and third lumbar vertebrae using CT histogram software (the “X section” analysis tool of Advantage Window 4.4; GE Healthcare). Second, the region of interest was placed as the outermost border of muscles using freehand manual drawing. Third, the area of these muscles ranging from -29 to 100 HU was calculated using CT histogram analysis. Then, the muscle mass was calculated as the cross-sectional area. In the case of the pectoralis muscle, the bilateral masses of the muscles were measured separately and the two values were averaged.

### Definition of sarcopenia

The muscle mass area was divided by height, and the values were reported according to the region of measurement as L3-SMI and PM-SMI (cm^2^/m^2^). Sarcopenia was defined as an SMI less than the muscle region- and sex-specific-cut-off values suggested by Prado et al. [34] and by our previous study [29] (L3-SMI: male, 52.4 cm^2^/m^2^; female, 38.5 cm^2^/m^2^; and PM-SMI: male, 44 cm^2^/m^2^; female, 31 cm^2^/m^2^). In this study, sarcopenia and non-sarcopenia were categorized as follows:

Sarcopenia-L3 – sarcopenia indexed by L3-SMI, regardless of PM-SMI (the rest are non-sarcopenia-L3);

Sarcopenia-L3 alone – sarcopenia indexed by L3-SMI with a non-sarcopenic level of PM-SMI;

Sarcopenia-PM – sarcopenia indexed by PM-SMI, regardless of L3-SMI (the rest are non-sarcopenia-PM);

Sarcopenia-PM alone – sarcopenia indexed by PM-SMI with a non-sarcopenic level of L3-SMI;

Sarcopenia-both – both L3-and PM-SMIs at sarcopenic levels;

Sarcopenia-L3/PM alone – sarcopenia-L3 alone or sarcopenia-PM alone;

Non-sarcopenia-both – neither L3-SMI nor PM-SMI at sarcopenic levels.

### Clinical data

Clinical data of patients were independently collected using electronic medical record review by two physicians (S-I Go and G-W Lee). Any discordant data were carefully discussed among the investigators. Baseline demographics, components of IPI, and other clinical findings of DLBCL were reviewed. Hypoalbuminemia was defined as serum albumin < 3.5 g/dL and elevated lactate dehydrogenase (LDH) as serum LDH > 225 IU/L. Body mass index (BMI) was calculated by dividing the weight in kilograms by the height in square meters (kg/m^2^). Underweight was defined as BMI < 18.5 kg/m^2^ according to Asian standards [43]. Treatment response was evaluated in available cases based on the revised International Working Group response criteria [44]. Treatment-related toxicity was assessed using the National Cancer Institute Common Toxicity Criteria (ver. 4.0) [45]. Treatment-related mortality (TRM) was defined as mortality caused directly by treatment at any time or as mortality resulting from any cause other than lymphoma progression within 30 days of the last cycle of R-CHOP. Treatment discontinuation was defined as a minimum of six cycles of R-CHOP for localized or advanced disease – and three to four cycles of R-CHOP with involved-field radiotherapy for localized disease – not being able to be performed.

### Statistical analysis

Comparisons between two groups were performed using the chi-square or Fisher's exact test for categorical variables and the Mann-Whitney U-test for continuous variables, as appropriate. For ordinal data with three categories (sarcopenia-both, sarcopenia-L3/PM alone, and non-sarcopenia-both), the chi-square test for trend and Kruskal-Wallis test were performed for categorical and continuous variables, respectively. Correlations between continuous variables were tested by Pearson's correlation coefficient. The median follow-up times were calculated by the reverse Kaplan-Meier method [46]. Overall survival (OS) was defined as the time from the beginning of treatment to death from any cause or last follow-up. PFS was defined as the time from the beginning of treatment to first progression, death from any cause, or last follow-up. Kaplan-Meier curves for survival data were plotted and compared via the log-rank test. Cox regression was performed to calculate the hazard ratio (HR) for death and disease progression along with its 95% confidence interval (CI). Potentially significant variables (*p* < 0.10) on univariate analysis were included in multivariate analysis. A value of *p* < 0.05 was considered to be statistically significant. All statistical analyses were conducted using Stata software (ver. 14.0; Stata Corp., College Station, Texas).
